# Surface Roughness Tuning at Sub-Nanometer Level by Considering the Normal Stress Field in Magnetorheological Finishing

**DOI:** 10.3390/mi12080997

**Published:** 2021-08-21

**Authors:** Xiaoyuan Li, Qikai Li, Zuoyan Ye, Yunfei Zhang, Minheng Ye, Chao Wang

**Affiliations:** Institute of Machinery Manufacturing Technology, China Academy of Engineering Physics (CAEP), Mianyang 621900, China; lxy20056482@126.com (X.L.); 13547806909@163.com (Q.L.); yebonpu@126.com (Z.Y.); zhangyf306@yeah.net (Y.Z.)

**Keywords:** magnetorheological finishing, surface roughness, force field simulation, normal stress

## Abstract

Although magnetorheological finishing (MRF) is being widely utilized to achieve ultra-smooth optical surfaces, the mechanisms for obtaining such extremely low roughness after the MRF process are not fully understood, especially the impact of finishing stresses. Herein we carefully investigated the relationship between the stresses and surface roughness. Normal stress shows stronger impacts on the surface roughness of fused silica (FS) when compared with the shear stress. In addition, normal stress in the polishing zone was found to be sensitive to the immersion depth of the magnetorheological (MR) fluid. Based on the above, a fine tuning of surface roughness (RMS: 0.22 nm) was obtained. This work fills gaps in understanding about the stresses that influence surface roughness during MRF.

## 1. Introduction

Fused-silica optics are integral to high-energy laser systems such as the wedged-focus and beam-sampling lenses at the National Ignition Facility (NIF) at Lawrence Livermore National Laboratory (LLNL) [1]. Producing fused silica optics with high-quality surfaces can reduce the scattering of laser energy and localize thermal focus, thus greatly enhancing the laser-induced damage threshold (LIDT) of the laser system [2,3]. Today, though the surface roughness of the fused silica can be improved to the sub-nanometer level via the chemical-mechanical polishing (CMP) [4,5], the frequent iteration of this method induces a low processing efficiency.

Magnetorheological finishing (MRF) is a novel kind of finishing technique that uses magnetorheological fluid consisting of carbonyl iron powder (CIP) in a basic liquid containing polishing grains [6,7]. Nowadays, MRF is widely considered to be a deterministic method to finish optics to an extremely high surface quality [8]. During the MRF process, a shear force dominates material removal; therefore, the relationship between the material removal rate (MRR) and force fields have been well investigated over the years [9,10,11,12]. However, the other important perspective for evaluating MRF, surface roughness, is seldom related to the force fields owing to the intrinsic complexity of the surface morphology evolution during MRF finishing. In previous reports, the improvement of FS surface roughness after MRF was mainly explained by mechanical and chemical theories from the CMP. Cumbo et.al. reported that smooth silica surfaces can be obtained by using small-sized polishing grains, and they emphasized the importance of the fluid chemistry [13]. Redien et. al. found that surface roughness did not depend on the slurry pH [4]. Generally, unlike the CMP process, in which a normal load dominated the material removal, in MRF the normal load was extremely low owing to the flexibility of the MRF fluid. In addition, the hardness or sizes of the polishing grains in the MRF fluid, the magnetic induction and size of the magnetic media, and the hydrodynamic behavior of the MRF fluid can be main factors that determine the surface roughness of the optics after finishing [14,15,16]. Ji et. al. found that a polymer-coated iron powder performed better in improving surface roughness of the FS compared with an uncoated powder since the soft polymer reduced the magnitude of the local stress imposed on the fused silica [7]. Li et. al. found a monotonous increasing non-linear correlation between subsurface damage depth and surface roughness because a larger normal load led to an expansion of surface or subsurface defects [17]. The abovementioned works indicated that when the composition of the MRF fluid was determined, the force fields in the polishing zone affected the surface roughness of the FS after the MRF process.

In this work, we evaluated the impact of force fields on surface roughness after MRF. A Reynolds equation based on hydrodynamic theory was used to resolve force distribution in MRF. By inputting the process parameters into theoretical calculation for an MRF experiment, the importance of shear and normal stress to the final FS surface roughness were compared. The discussion validated the stronger effect of normal stress on surface roughness compared to shear stress.

## 2. Methodology

Realistic MRF experiments were conducted together with simulation experiments. For each round of single-factor experiments, only one process parameter was changed. These parameters were immersion depth, polishing fluid viscosity and rotational speed. Each round of the polishing experiment was conducted by the same numerical control program of the machine tool. The water-based polishing fluid contained carbonyl iron powder, polishing grains and other stabilizers. The carbonyl iron particles (CIPs), about 2 µm, were the HF type purchased from the BASF SE. The other additives were mainly composed of ceria abrasives, de-ionized water, and Methoxypolyethylene glycols (MPEGs). The work being finished was on a piece of fused silica (FS), the initial surface roughness of which was 0.72 nm as measured by the Talysurf CII white light interferometer. For each determined setting of the process parameters, the experiments were repeated two times to reduce accidental errors. In addition, to obtain the MRF removal function, the MR polishing ribbon interacted with the FS workpiece for 6 s. The rotational speed of the polishing wheel were set at 80–110 rpm; the immersion depth of the finishing fluid was set at 0.2–0.5 mm, the viscosities of the MRF fluid were 144, 155, 174, and 190 Pa. s. A straight line removal mode of the removal function was used to obtain a super-smooth FS surface.

**Figure 1:**
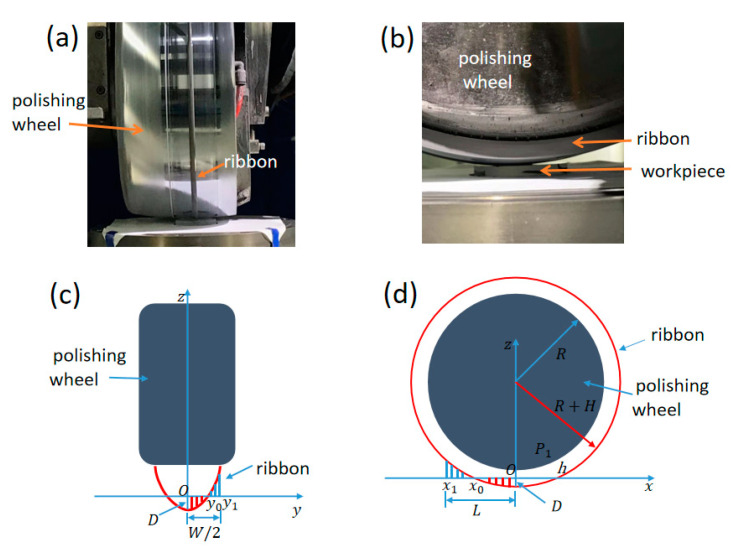
(**a**) MRF fluid ribbon on the polishing wheel, (**b**) contact of the fluid ribbon and the workpiece, (**c**) coordinate system of the polishing wheel and workpiece seen from the *x*-axis and (**d**) coordinate system of the polishing wheel and the FS work-piece seen from the *y*-axis.

Owing to the non-Newtonian characteristic of the MR fluid, the normal and shear stress fields in the polishing zone were obtained by resolving the two-dimensional Reynolds equation based on hydrodynamic theory [18,19]. The coordinate system of the MRF machine is presented in Figure 1c,d. Based on the classic hydrodynamic lubrication derivation process [18], we derived the Reynolds equation of the MRF coordinate system as

(1) $$\frac{\partial }{\partial x}\left(\frac{h^{3}}{\eta }\frac{\partial p}{\partial x}\right)+\frac{\partial }{\partial y}\left(\frac{h^{3}}{\eta }\frac{\partial p}{\partial y}\right)=6U\frac{\partial h}{\partial x}$$

Speed distribution is expressed by Equation (2), in which *U*_2_ represents the velocity of the rotational wheel and *U*_1_ is the speed of the MRF fluid. Now, Equation (1) can be resolved through the finite difference method, the boundary conditions for which are set as Equations (3) and (4) where *s* is the finishing area, *n* is the vector of the *z* axis, h is the distance from the workpiece surface to the bottom of the polishing wheel, and H is the thickness of the MRF fluid ribbon. A mid-difference method was used to resolve the pressure stress *p* (*x*,*y*) distribution.

(2) $$u=\frac{1}{2\eta }\frac{\partial p}{\partial x}\left(z^{2}-zh\right)+\left(U_{2}-U_{1}\right)\frac{z}{h}+U_{1}$$

(3) $${p|}_{s}=0$$

(4) $${\frac{\partial p}{\partial n}|}_{s}=0$$

After inputting the process parameters (immersion depth, viscosity, and polishing wheel rotational speed) into the Reynolds equation, iterations resolved the normal stress *p.* Finally, by calculating the partial differential of the normal stress, the shear stress was obtained.

**Figure 2:**
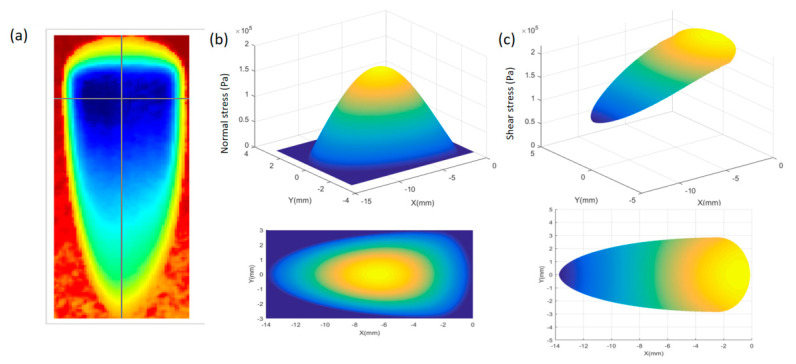
(**a**) MRF polishing spot measured by a ZYGO laser interferometer, (**b**) calculated normal stress distribution, (**c**) calculated shear stress distribution: (top: three-dimensional view of the polishing spot or stress distribution, bottom: view from the *z* axis of the coordinate system of the workpiece).

In general, the supersmooth FS surface was obtained by iteration of the removal function; in other words, as one polishing spot moving along a determined pathway, the whole surface of the FS was finished. Figure 2a shows six typical D-like shape-removal functions on an FS workpiece under various parameters after the MRF spot taking tests. Figure 2b,c shows the simulated normal and shear stress fields calculated by Equation (1). The “D-like” shape of the stress fields was inconsistent with the real removal function of the MRF experiment in Figure 2a. In addition, the orders of the stress magnitude were calculated at a kPa level, which was close to previous measurements [11,20].

## 3. Results and Discussion

**Figure 3:**
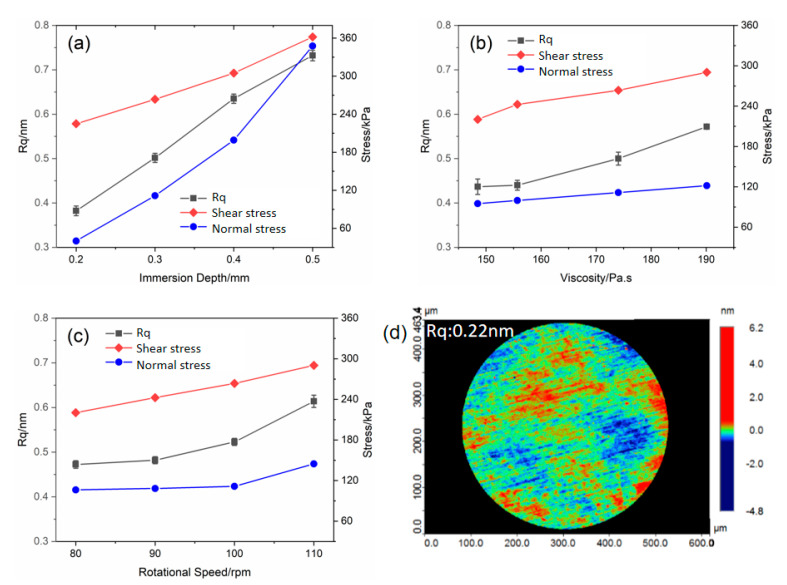
Relationships between surface roughness/peak shear stress/peak normal stress and process parameters including (**a**) immersion depth, (**b**) viscosity of MR fluid and (**c**) rotational speed. (**d**) Surface roughness of the FS under the determined process parameter.

Figure 3a shows that when the immersion depth was 0.2 mm, the surface roughness (Rq) of the FS was reduced from 0.72 to 0.31 nm. Increasing the immersion depth severely deteriorated the final surface quality. At an immersion depth of 0.5 mm, the surface roughness only improved from 0.75 to 0.72 nm. In an immersion depth range of 0.2–0.5 mm (not large for practical MRF) the final Rq saw a dramatic enhancement from 0.31 to 0.72 nm, which indicated that surface roughness after MRF was sensitive to immersion depth.

Figure 3b shows that as the viscosity of the MR fluid increased from 155 to 190 Pa. s, the final Rq changed slightly from 0.48 to 0.68 nm. Such a phenomenon indicated that increasing viscosity could lead to the deterioration of surface quality. In addition, further decreasing the viscosity (148 Pa. s) did not obviously change surface quality (Rq: 0.44 nm).

Regarding rotational speed, as it increased from 90 to 110 rpm as shown in Figure 3c, the corresponding Rq changed from 0.48 to 0.60 nm. To achieve better surface quality, we reduced the speed to 80 rpm; however, the Rq was only reduced to 0.47 nm. It is clear that with the enhanced rotational speed, the shear stress followed a linear trend. In contrast, normal stress increased exponentially similar to the Rq curve.

The abovementioned phenomena indicated that when the rotational speed or viscosity of the MR fluid fell below a liminal value in the only-one-factor changed MRF experiment, the surface roughness of the FS did not visibly improve, whereas other process parameters were more dominant.

Li et. al. reported the monotonously increasing non-linear correlation between surface roughness and the density of the subsurface defect, which mainly originated from the normal load [18]. Then the correlation of stress fields and surface roughness was carefully considered. Specifically, as the immersion depth increased from 0.2 to 0.5 mm, the peak normal stress, defined as the largest normal stress in the entire polishing zone, saw a dramatic change from 42 to 353 kPa; meanwhile, the peak shear stress rose slightly (238 to 360 kPa). Such results indicated that peak normal stress was more sensitive than peak shear stress to the immersion depth.

With the enhancement of MR fluid viscosity, the peak normal stress changed from 95 to 123 kPa as shown in Figure 3b; meanwhile, the peak shear stress saw a 230 to 290 kPa enhancement. In contrast to immersion depth, the increase of the stresses were not obvious as shown in Figure 3a. A similar tendency was also found for the rotational speed. As the polishing wheel increased from 80 to 110 rpm, the peak normal only changed from 107 to 145 kPa (peak shear stress: 232 to 285 kPa). From Figure 3a–c, a lower immersion depth, viscosity, and rotational speed led to an ultrasmooth finished FS surface. In a modified process parameter setting, surface roughness can be reduced to 0.22 nm as shown in Figure 3d.

**Figure 4:**
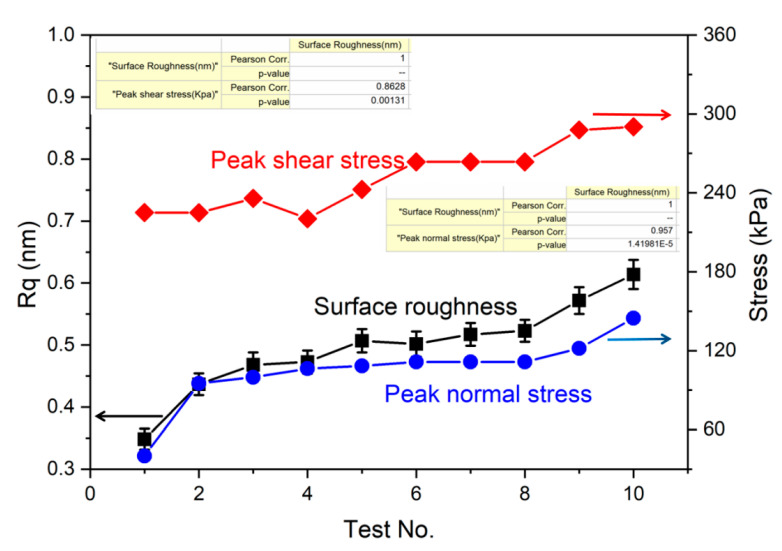
Comparison of the stresses and surface roughness (Rq) in 10 rounds of the MRF experiments.

To gain a deeper insight into the relationship between surface quality and the imposed stresses on the FS workpiece, we compared the Rq and the peak stresses of each MRF test. The *x* coordinate represents the stresses, and the *y* coordinate represents the Rq. In Figure 4, a parallel-like tendency of the Rq and peak normal stress was detected. In comparison, the shear stress showed a slight fluctuation, and the correlation between the shear stress and the Rq was not close.

(5) $$r=\frac{N\sum x_{i}y_{i}-\sum x_{i}\sum y_{i}}{\sqrt{N\sum x_{i}^{2}-{\left(\sum x_{i}\right)}^{2}}\sqrt{N\sum y_{i}^{2}-{\left(\sum y_{i}\right)}^{2}}}$$

In a quantitative evaluation model, the Pearson correlation coefficients of peak normal stress and shear stress to surface roughness was calculated to be 0.967 and 0.862, respectively. The Pearson correlation coefficient is depicted in Equation (5). When r is closer to 1, *x* and *y* are more correlated. Obviously, the stronger impact of the normal stress compared with the shear stress on the final surface roughness after MRF was re-identified.

## 4. Conclusions

Overall, the relationship between the stresses in the polishing zone and the surface roughness after magnetorheological finishing was studied. Specifically, the surface roughness was found to be much more correlated to peak normal stress than with peak shear stress. The correlation coefficients of the normal stress and shear stress to surface roughness were 0.967 and 0.862, respectively. Increasing the immersion depth, viscosity of the MRF fluid, and polishing wheel rotational speed contributed to a larger normal stress, which led to deterioration of the surface roughness in the MRF process. Such results helped us predict the surface roughness under specific process parameters, thereby reducing experimental trials to obtain a better surface quality of fused silica, which leads to time saving and lower costs. Furthermore, the correlation between the surface roughness and peak normal stress may provide insight into the mechanisms of fused silica surface roughness after MRF.
